# Enhanced trajectory tracking and robustness in magnetic levitation via takagi-sugeno fuzzy control: experimental approach

**DOI:** 10.1038/s41598-025-25356-y

**Published:** 2026-01-07

**Authors:** Yuvapriya T, Vimala Kumari Jonnalagadda, Vijaya Lakshmi Korupu, Vinodh Kumar Elumalai

**Affiliations:** 1 School of Electrical Engineering, Vellore Institute of Technology, Vellore, Tamil Nadu 632014 India; 2 Department of EEE, Siddhartha Academy of Higher Education, Deemed to be University, Vijayawada, Andhra Pradesh 520007 India; 3 Department of EIE, Siddhartha Academy of Higher Education, Deemed to be University, Vijayawada, Andhra Pradesh 520007 India

**Keywords:** Engineering, Mathematics and computing, Physics

## Abstract

Robust control of magnetic levitation (maglev) plant remains a significant challenge due to its inherent non-linearities and uncertainty to exogenous perturbations, though maglev technology has a wide range of usages, from high-speed trains to advanced robotics. To solve these problems and improve the maglev system’s trajectory-tracking performance and robustness, this research proposes a control technique that involves synthesizing a T-S fuzzy controller using the parallel distributed compensation (PDC) method. The controller design is further augmented with a velocity-compensation technique to enable smooth and frictionless ball levitation in a maglev system. The gravitational bias acting on the maglev system is controlled by integrating the feed-forward controller ($F_f$) with the PDC-TS fuzzy scheme. The Lyapunov function candidate and linear matrix inequalities (LMIs) are explored to determine the proposed TS fuzzy scheme’s global asymptotic stability. Finally, the effectiveness of the control technique is experimentally evaluated for several test cases using hardware-in-loop (HIL) testing on the maglev system. The results corroborate that the T-S fuzzy control strategy offers robustness and trajectory tracking of the system with stable levitation over the traditional PIV scheme.

## Introduction

Magnetic levitation (maglev) technology has acquired prominence in tracking control applications due to its unique ability to suspend and move objects without physical contact. This technology eliminates friction, offering a smooth and efficient method for transportation and positioning in various applications, such as high-speed trains, advanced manufacturing processes, and even precision medical devices^1,2^. Whether in semiconductor manufacturing, laboratory automation, or advanced robotics, maglev tracking systems excel in providing smooth, frictionless movement and eliminating mechanical wear, thereby enhancing reliability and longevity^3^. With their ability to operate in various environments, withstand harsh conditions, and adapt to diverse load requirements, maglev tracking solutions are represent cutting-edge technologies, propelling industries toward greater efficiency and innovation. Designing an effective controller is a significant task due to inherent non linearity and instability of maglev systems in open-loop settings. Researchers have addressed this challenge through various control techniques documented in the literature, aiming to achieve robust and precise tracking control. Various control strategies have been devised, including input–state and input-output linearization approaches^4,5^. Additionally, absolute eigenvalue approach^6^, linear state feedback controls^7^, observer-based controls^8,9^, cognitive techniques^10,11^, sliding mode control^12,13^, backstepping control^14^, model predictive control (MPC)^15,16^, LQR control^17^ and Iterative Learning Control^18^ have been explored in controller design.

PID controllers require a thorough understanding of plant dynamics to function properly, and any variation between the predicted model and the actual system might result in poor control. Maglev systems, being inherently complex and nonlinear, may exhibit dynamics that are challenging to capture precisely in a model. Furthermore, the impact of periodic disturbances poses an additional challenge for conventional PID controllers. Maglev systems, like many other real-world applications, are often subject to periodic disturbances that can significantly affect their performance. The fixed and predetermined nature of PID control parameters may not be well-suited to handle the dynamic nature of these disturbances. The inherent adaptability of PID controllers is limited, and their response to periodic disturbances might lead to sub-optimal control, oscillations, or even instability in the system. Though feedback controllers deal a significant amount of model variation, their performance is always limited by the “waterbed effect”. The principle states that increment in system’s bandwidth to better tracking and disturbance rejection would eventually raise the sensitivity to high-frequency oscillations. Despite the notable research findings of the existing works, which have not been thoroughly studied, we develop a fuzzy logic architecture to better the tracking performance and robustness of the system over external perturbations and model uncertainty.

Fuzzy Logic Control (FLC) proves advantageous for ball tracking control in maglev systems due to its inherent ability to handle the nonlinear and uncertain dynamics associated with such systems. In the context of a maglev system tracking a ball, factors like air gap, variations in ball mass, unpredictable external forces such as changing temperatures and initial conditions of the system can affect the system behaviour. As a result, a T-S fuzzy model is developed using PDC framework with the incorporation of the if/then rules and the velocity compensation method. The essential part of the PDC is to provide a strategy for creating a fuzzy controller from a given T-S fuzzy model^19^. Initially, the PDC technique divides a nonlinear system into several linear subsystems. Then, for every linear subsystem, a separate controller is developed. These local controllers would eventually be combined to create an equivalent fuzzy controller to meet the necessary control goals. The key reason for utilizing TS fuzzy control is that it eliminates the need for an exact dynamical model. Moreover, the adaptive nature of TS fuzzy control enables the controller to learn from input and output data and adapt to changing dynamics with fewer rules, even for highly non-linear and uncertain systems. This flexibility results in precise and responsive control, ensuring effective ball tracking even in uncertain situations. T-S fuzzy control frameworks are increasingly being used for complex real-time systems because of their capacity to handle parameter uncertainties and external disturbances while offering model-based stability guarantees^20^. Fuzzy logic control for effective mobile robot path planning in both static and dynamic interior situations^21^ and improved cascade control for stable levitation of maglev trains^22^, demonstrate the broad applicability of fuzzy-based nonlinear control techniques. Since magnetic levitation (maglev) systems have been used in advanced robotic manipulation, semiconductor handling, high-speed transit, and medical device positioning, accurate trajectory tracking and robustness under external disturbances are the metrics employed for assessing the potential of the TS fuzzy controller. The PDC-TS fuzzy control approach suggested in this study directly meets these needs and offers a scalable and reliable solution appropriate for real-world maglev applications.The findings of the work demonstrate the possibilities of T-S fuzzy for supervising complex, non-linear, and uncertain plants, which improves control strategies for magnetic levitation systems. Furthermore, it can avoid the convergence issue related to membership function adjustments and enhance tracking performance. In this regard, the significant contributions are listed below.

1. To define the global performance of a nonlinear system, a PDC TS fuzzy model is constructed. We then incorporate a velocity compensation method to realize a feasible control framework. This model is ideal for ball tracking control in maglev systems because it can naturally handle the nonlinear and uncertain dynamics as well as the fuzzy convergence issue. The velocity compensation strategy minimizes undesired bumps in the control signal caused by rapid changes in the set point.
2. The system’s stability is assessed using the Lyapunouv method.
3. The PDC-TS fuzzy control method’s robustness and performance are confirmed through HIL testing on a maglev system.
4. Three test scenarios are presented to validate the performance against exogenous disturbances and uncertainties, which include nominal reference tracking, command tracking under external disturbance, and model uncertainty.
5. A CPSD analysis is presented to evaluate tracking control performance in both transient and steady-state scenarios over the spectrum of frequency.

The remnant of the paper is arranged as follows. Section 2 establishes the problem formulation in the context of the T-S fuzzy framework. Section 3 describes the magnetic levitation mechanism about Newton’s force balance equation. Section 4 gives a detailed explanation of the stability utilizing the Lyapunov function candidate. Section 5 contains a full assessment of the advantages and efficacy of FLC in the context of tracking the position of a maglev ball, the hardware setup and results. In this end, Section 6 contains the paper’s conclusion comments.

## Problem formulation

A nonlinear T-S fuzzy system is expressed as

1 $$\begin{aligned} \begin{aligned} R^i:IF \hspace{5.0pt}{q}_1(t) \hspace{5.0pt}is \hspace{5.0pt}{M_1}^{i} \hspace{5.0pt},..., \hspace{5.0pt}{q}_n(t)&\hspace{5.0pt}is \hspace{5.0pt}{M_n}^{i} \hspace{5.0pt}THEN \\&{\left\{ \begin{array}{ll} {\dot{x}}{(t)}=A_ix(t)+B_iu(t)\\ y(t)=C_ix(t)\\ x(0)=x_0 \end{array}\right. } \end{aligned} \end{aligned}$$

where R represents the fuzzy inference using r fuzzy rules, $q_n$ indicates the premise variables of input fuzzy set. ${M_j}^{i}$ represents the input fuzzy set, $j=1,2,\dots ,n$. The system state, input, and output matrices are $A_i\in \mathbb {R}^{l*l}$, $B_i\in \mathbb {R}^{l*m}$, and $C_i\in \mathbb {R}^{p*l}$. The state, input, and output vectors are represented as *x*(*t*), *u*(*t*), and *y*(*t*) $\in \mathbb {R}^l, \mathbb {R}^m$, and $\mathbb {R}^p$. The matrices associated with the system are given by,

$$A= \begin{bmatrix} 0& 1\\ {w_b}^2 & 0 \end{bmatrix}$$, $$B=\begin{bmatrix} 0\\ 1 \end{bmatrix}$$, and $$C=\begin{bmatrix} 0&{-{k_b}{w_b}^2} \end{bmatrix}$$. The origin is assumed to be the equilibrium of the system (1). Denote

2 $$\begin{aligned} \mu _{{R_i}}(q(t))=\prod \limits _{i=1}^{r}\prod \limits _{j=1}^{n} {M_j}^{i} (q_j(t)) \end{aligned}$$

The grade membership of $q_j(t)$ is denoted as ${M_j}^{i} (q_j(t))$, which satisfies the condition below,

$$\begin{aligned} \sum _{i=1}^{r} \mu _{R_i}(q(t))\ge 0 \end{aligned}$$

let,

3 $$\begin{aligned} h_i(q(t))=\frac{\mu _{R_i}(q(t))}{\sum _{i=1}^{r}\mu _{R_i}(q(t))} \end{aligned}$$

Therefore, $h_i(q(t))\ge 0$ $\forall$ t, and $\sum _{i=1}^{r}h_i(q(t))=1$. $h_i(q(t))$ $\in \hspace{5.0pt}[0,1]$ indicates the normalized membership function. The T-S fuzzy model, in 1, is defined as

4 $$\begin{aligned} \begin{aligned}&\dot{x}(t)=A(\mu )x(t)+B(\mu )u(t)\\&y(t)=C(\mu )x(t) \end{aligned} \end{aligned}$$

where, $A(\mu )=\sum _{i=1}^{r}h_i(q(t))A_i$, $B(\mu )=\sum _{i=1}^{r}h_i(q(t))B_i$, and $C(\mu )=\sum _{i=1}^{r}h_i(q(t))C_i$. The control goal involves to generate a control signal *u*(*t*) using a state feedback rule. This *u*(*t*) guarantees the convergence of the measured output *y*(*t*) to vicinity of the reference signal $y_d(t)$.

5 $$\begin{aligned} IF \hspace{5.0pt}{q}_1(t) \hspace{5.0pt}is \hspace{5.0pt}{M_1}^{i} \hspace{5.0pt},..., \hspace{5.0pt}{q}_n(t)\hspace{5.0pt}is \hspace{5.0pt}{M_n}^{i} \hspace{5.0pt}THEN\hspace{5.0pt}\hspace{5.0pt}u(t)=T_ix(t) \end{aligned}$$

where, $T_i$ is the state feedback controller parameter associated with $i^{th}$ rule. Let,

6 $$\begin{aligned} \begin{aligned}&u(t)=\sum _{i=1}^{r}h_i(q(t))T_ix(t)\approx T(\mu )x(t)\\ and\\&T(\mu )=\sum _{i=1}^{r}h_i(q(t))T_i \end{aligned} \end{aligned}$$

Substitute 6 into 4, which yields

7 $$\begin{aligned} \begin{aligned}&\dot{x}(t)=A(\mu )x(t)+B(\mu )T(\mu )x(t)\\&y(t)=C(\mu )x(t) \end{aligned} \end{aligned}$$

**Assumption 1.** For any desired trajectory $y_d$, there exists $u_d$ such that

8 $$\begin{aligned} \begin{aligned}&{\left\{ \begin{array}{ll} {\dot{x}}_d{(t)}=A(\mu )x_d(t)+B(\mu )T(\mu )x_d(t)\\ y_d(t)=C(\mu )x_d(t)\\ x_d(0)=x_0 \end{array}\right. } \end{aligned} \end{aligned}$$

Fuzzy control optimizes the controlling effort of the system (1) that produces the output, *y*(*t*), to eventually achieve the command trajectory $y_d(t)$ $\forall$t $\in$ $[0, T_f]$.

$$\lim \limits _{t\rightarrow \infty }u(t)=u_d(t)$$   $$\implies$$ $$\lim \limits _{t\rightarrow \infty }y(t)=y_d(t)$$

Thus, the tracking error $e(t)=y_d(t)-y(t)$ decreases monotonically.

$$\lim \limits _{t\rightarrow \infty }e(t)=0$$

## Magnetic levitation plant

**Fig. 1:**
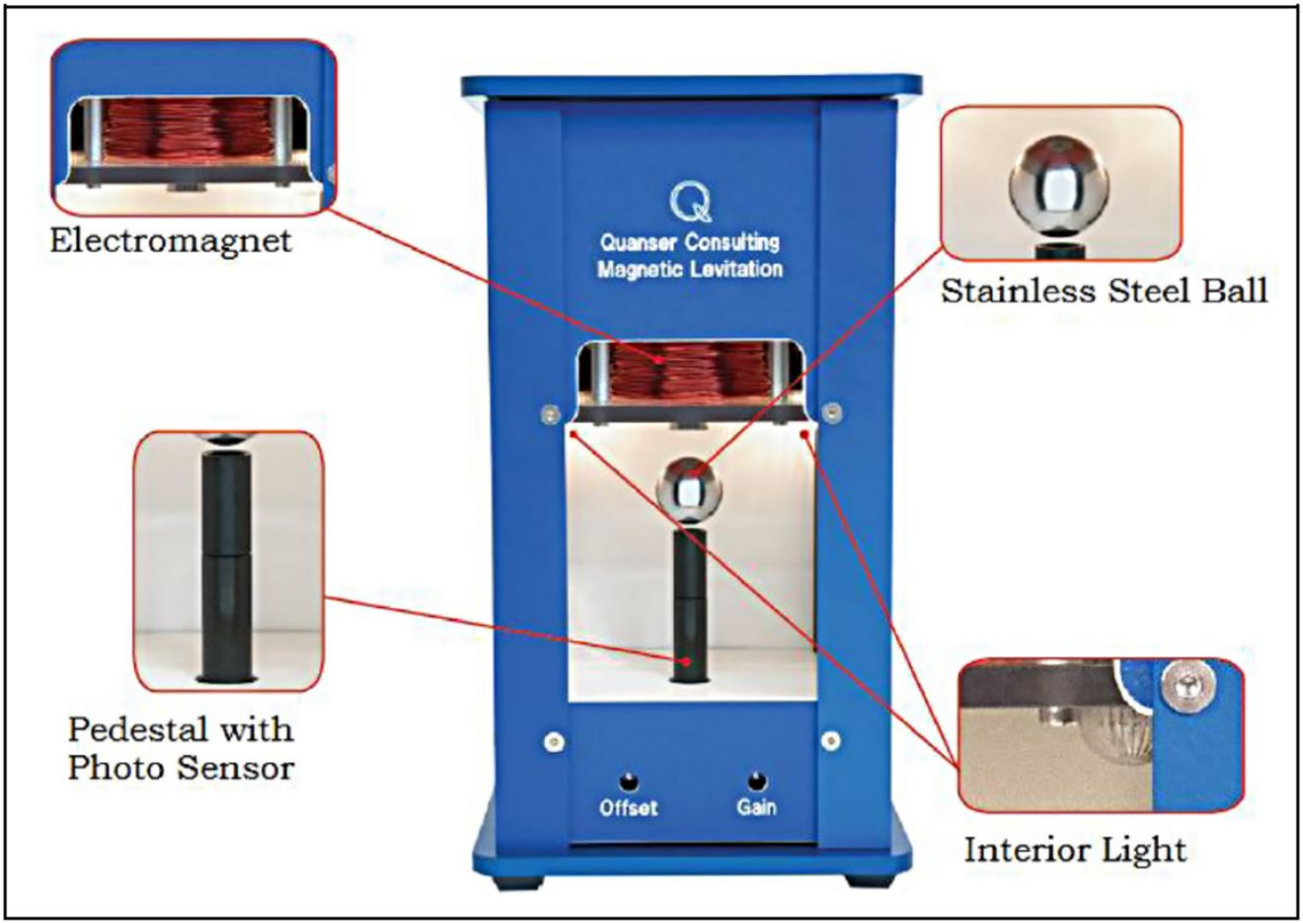
Magnetic levitation plant.

Figure 1 depicts the conventional maglev plant, levitating a steel ball in the air while it is within its magnetic field. The maglev is comprised of three distinct portions that are encased within a rectangular enclosure. In its upper section, there is an electromagnet fabricated with a solenoid steel core. There is an inner chamber in the centre of the section where the magnetic ball suspension occurs. A photo sensor is situated at the lower area of the maglev plant and is integrated into the post to detect the location of the ball. The goal is to suspense the ball in mid-air as well as to follow the command signal via modulating the electromagnet coil current. The maglev parameters are given in Table 1.

**Table 1:** Maglev plant parameters.

**Symbol**	**Description**	**Value**	**Unit**
$L_c$	Coil Inductance	412.5	mH
$R_c$	Coil Resistance	10	$\Omega$
$l_c$	Coil Length	0.0825	m
$R_s$	Current sensing resistance	1	$\Omega$
$r_b$	Ball Radius	1.27 $\times {10^{-2}}$	m
$M_b$	Ball Mass	0.068	kg
$T_b$	Ball Travel	0.014	m
*g*	The Gravitational Coefficient	9.81	$m/s^2$
$x_{b0}$	Equilibrium position	0.006	m
$i_{C0}$	Equilibrium current	0.86	A
$K_B$	The sensitivity of the ball position sensor	2.83$\times {10^{-3}}$	m/V
$K_m$	Electromagnetic force constant	6.5308$\times {10^{-5}}$	$N-m^2/A^2$

### Mathematical modeling

**Fig. 2:**
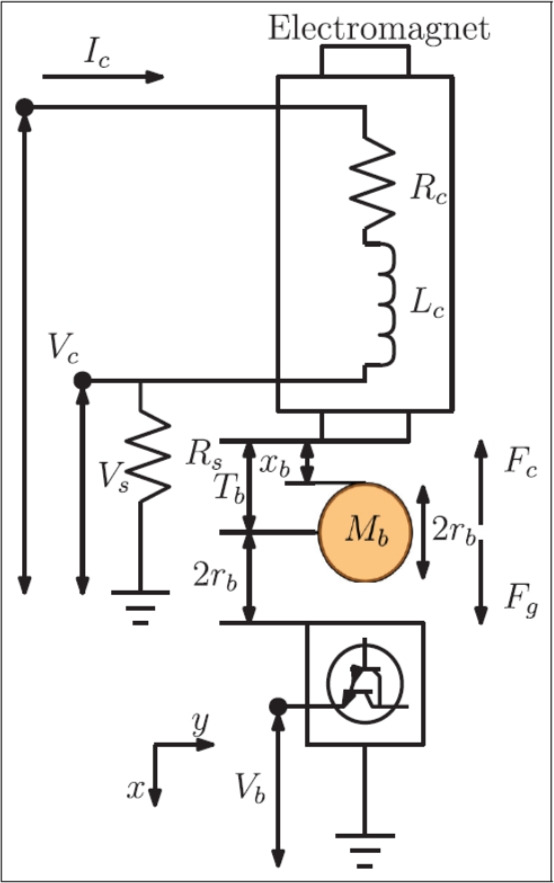
maglev schematic representation.

An illustration of the maglev plant can be found in Fig. 2. The ball has six degrees of freedom (DOF), however, the vertical direction is only controlled. The maglev is unstable and under-actuated in an open loop, developing a controller is difficult. According to the illustration, the electromagnet force $F_c$ subjected to the ball is defined as follows

9 $$\begin{aligned} F_c=\frac{K_m{I_c}^2(t)}{2{x_b}^2(t)} \end{aligned}$$

where $K_m$, $I_c$, and $x_b$ represent the electromagnetic force constant, coil current, and ball actual position, respectively. The ball’s gravitational force $F_g$ is given by

10 $$\begin{aligned} F_g=M_bg \end{aligned}$$

The total external force exerted to the ball can be calculated as

11 $$\begin{aligned} F_{ext}=-F_c+F_g=-{\frac{K_m{I_c}^2(t)}{2{x_b}^2(t)}}+M_bg \end{aligned}$$

From Newton’s second law, we get

12 $$\begin{aligned} M_b\ddot{x_b}(t)=-{\frac{K_m{I_c}^2(t)}{2{x_b}^2(t)}}+M_bg \end{aligned}$$

The nonlinear equation is expressed as

13 $$\begin{aligned} \ddot{x_b}(t)=-{\frac{K_m{I_c}^2(t)}{2M_b{x_b}^2(t)}}+g \end{aligned}$$

Using Taylor’s series to linearize equation (12) at nominal operating regions $(x_{b0}, I_{c0})$, the system transfer function can be obtained

14 $$\begin{aligned} G(s)=\frac{\Delta x_b(s)}{\Delta I_c(s)}=\frac{-K_b{\omega _b}^2}{s^2-{\omega _b}^2} \end{aligned}$$

where $\omega _b = \sqrt{\frac{2g}{x_{b0}}}$ and $K_b={\frac{x_{b0}}{I_{c0}}}$ are the oscillation frequency and the steady-state gain, respectively. Due to its inherent nonlinearities and instability, the system frequently deviates from the feasible tracking performance, even when linearized at the nominal operating point. In this regard, we present the TS fuzzy in combination with a traditional feedback control technique to ensure the better tracking control of a maglev plant.

## TS-fuzzy controller for tracking and robustness

**Fig. 3:**
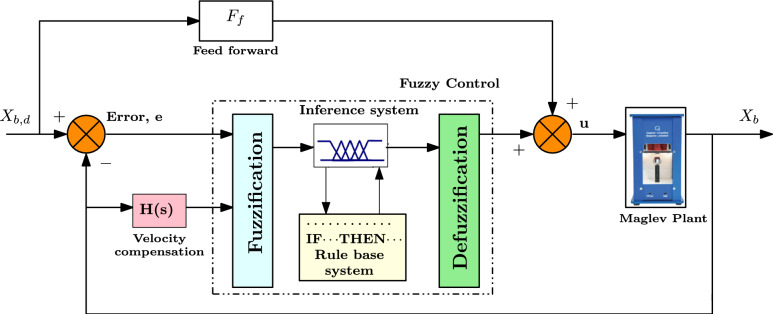
TS fuzzy control scheme for maglev system.

Figure 3 depicts the TS fuzzy approach for the maglev plant. The fuzzy controller receives the fuzzy inputs such as position error (e) and ball velocity $(dx_b/dt)$, and outputs a precise coil current $(I_c)$ to regulate $x_b$. The proposed structure prevents the “derivative kick” by using ball position velocity as an input, unlike standard fuzzy systems that employ error velocity as an input membership function. Velocity error is primarily used in the feedback to prevent the deviation of ball position from the point of interest. A Velocity correction module with frequency $w_f$=15.7 rad/sec is considered to alter the measurement noise.

15 $$\begin{aligned} H(s)=\frac{w_fs}{s+w_f} \end{aligned}$$

The T-S or Type-III fuzzy model depicts the system dynamics into linear models with fuzzy membership functions. Surprisingly, Fuzzy T-S models are approximators for universal functions that may be used to any convex compact surface and can precisely approximate any smooth nonlinear function to any desired degree^23^. Furthermore, according to^24^, Fuzzy control based on the TS model offers methodical techniques for stability investigation and controller design. Consider the input vector is,

16 $$\begin{aligned} q= \begin{bmatrix} q_1 \\ q_2 \end{bmatrix}= \begin{bmatrix} e \\ \frac{dx_b}{dt} \end{bmatrix} \end{aligned}$$

The bounds on *e* and $(dx_b/dt)$ are $q_1$ $\in$ [-0.3 0.3] mm, $q_2$ $\in$ [-1.2 1.2] mm/s. The range of coil current required to smoothly control the coil voltage is equivalent to $I_c$ $\in$ [-2 2] A. Fuzzification takes into account the following five fuzzy labels: Positive Big (PB), Positive Small (PS), Zero (Z), Negative Small (NS), and Negative Big (NB). $M_j^i$ and $N_1^i$ are the input and output fuzzy sets, and every input belongs to at least one of these sets. The Gaussian technique is applied to represent the membership of each input-output fuzzy set.

17 $$\begin{aligned} & \mu _{M_j^i}(q)=exp^{-\frac{\left( q-C\right) ^2}{2{(\sigma )^2}}} \end{aligned}$$

18 $$\begin{aligned} & \mu _{N_1^i}(I_c)=exp^{-\frac{\left( {I_c}-C\right) ^2}{2{(\sigma )^2}}} \end{aligned}$$

The mean of the fuzzy set is denoted by ‘*C*’, its standard deviation by ‘$\sigma$’, and its input and output membership functions are denoted by $\mu _{M_j^i}$ and $\mu _{N_1^i}$, respectively. Therefore, the input and output fuzzy sets are,

19 $$\begin{aligned} & M_n^i=\{(q, \mu _{M_n^i}(q)):q_1\in [-0.3\hspace{5.0pt}0.3], q_2\in [-1.2\hspace{5.0pt}1.2]\} \end{aligned}$$

20 $$\begin{aligned} & N_1^l=\{(\theta , \mu _{N_1^l}(\theta )):\theta \in [-2\hspace{5.0pt}2]\} \end{aligned}$$

Table 2 shows the output and input Gaussian membership distinctive values, whereas Table 3 displays the 5*5 fuzzy rules. While heuristic considerations influenced the initial selection of fuzzy rules and membership functions, the controller synthesis was then conducted systematically using the PDC framework with Lyapunov-based LMI constraints to guarantee theoretical stability and performance. Note that the proposed controller does not use the Mamdani structure, which makes use of fuzzy set outputs and centroid-based defuzzification, but rather the T-S framework, where the rule consequents are linear state-feedback gains integrated via normalized membership functions.

**Table 2:** Properties of membership functions.

MF’s	e		$dx_b/dt$		$I_c$	
	$\sigma$	c	$\sigma$	c	$\sigma$	c
NB	0.064	-0.3	0.254	-1.2	0.425	-2
NS		-0.15		-0.6		-1
Z		0		0		0
PS		0.15		0.6		1
PB		0.3		1.2		2

**Table 3:** Fuzzy rule table.

${dx_b/dt}$	e				
	NB	NS	Z	PS	PB
NB	PB	PB	PS	PS	Z
NS	PB	PS	PS	Z	NS
Z	PB	PS	Z	NS	NB
NS	PS	Z	NS	NS	NB
NB	Z	NS	NS	NB	NB

The sum of input fuzzy sets and their firing intensity determines the output fuzzy set. For example, the ‘*AND*’ operator in the provided rule structure is used to apply the fuzzy inputs, and it also determines the firing strength by evaluating the smallest or combined value of the membership functions of the input for each rule^25^. For the rule $i^{th}$, the truth level of the input fuzzy set

21 $$\begin{aligned} \mu _{R_i}=\{ \mu _{M_1^i}(q_j) \hspace{5.0pt}\mu _{M_2^i}(q_j):q_j\in q,j=1,2\dots ,n\} \end{aligned}$$

where ‘$\mu _{R_i}$’ represents the membership function firing strength associated with $i^{th}$ rule. The controller output is defuzzified using the centre of gravity (COG) procedure.

22 $$\begin{aligned} y=\frac{\Sigma _{i=1}^{25}\mu _{R_i} y_i d(y_i)}{\Sigma _{i=1}^{25}\mu _{R_i}} \end{aligned}$$

**Fig. 4:**
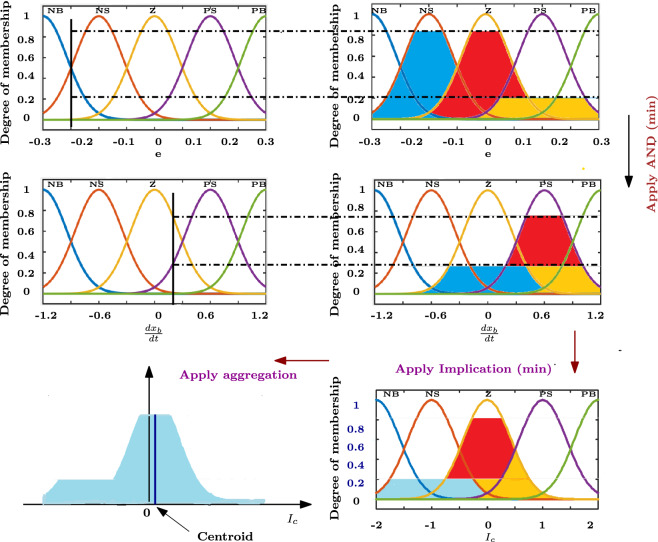
Fuzzification and defuzzification Process.

**Fig. 5:**
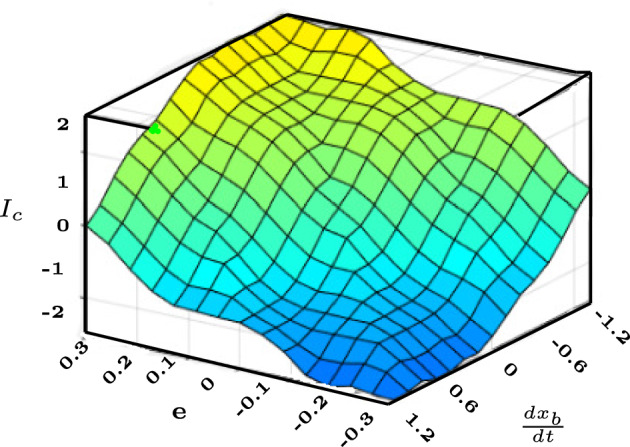
Surface Plot.

Figure 4 shows the complete fuzzy logic process where the total area is partitioned into smaller pieces and the fuzzy output is defuzzified to the crisp value using the COG technique. Figure 5 depicts the control surface plot, which provides the local and global features of the fuzzy controller and presents the impact of the two inputs on the output. The surface plot also shows that coil current is regulated as required to maintain the threshold limits of the ball velocity as the error increases.

The PDC fuzzy, which uses similar condition variables to the T-S framework, has three steps. First, the nonlinear dynamics are separated as a collection group of linear models. Second, a linear controller is constructed for every local linear system using the blending approach and combined to generate the overall controller. Finally, the LMI analysis is employed to evaluate the stability of the fuzzy closed-loop system^26^. The Lyapunov direct approach with LMI constraints is used to show that the T-S fuzzy system is stable.

### Theorem 1

As stated in (1), consider the system with a stabilizing controller and its fuzzy rules $R^k$. When the Lyapunov function derivative is non-increasing, that is, $\dot{V}(x)\le 0$, the nonlinear system is stable asymptotically at the origin $x_0$ such that there is a continuously differentiable positive function

$$V:R^n\rightarrow \mathbb {R}$$ ,$$V=(x(t))^TP(x(t))$$

where $P \in \mathbb {R}^{n \times n}$ denotes the symmetric positive definite matrix with $PF_i+F^T_iP< 0$.

### **Proof**

The Lyapunov time derivative is,

23 $$\begin{aligned} \dot{V}(x)={(\dot{x}(t))}^TP(x(t))+(x(t))^TP{(\dot{x}(t))} \end{aligned}$$

Substitute (4) into (23),

24 $$\begin{aligned} \begin{aligned} \dot{V}(x)&=\left[ A(\mu )x(t)+B(\mu )u(t)\right] ^TP(x(t))\\&+(x(t))^TP\left[ A(\mu )x(t)+B(\mu )u(t)\right] \end{aligned} \end{aligned}$$

The rearrangement of equation (24) is

25 $$\begin{aligned} \begin{aligned} \dot{V}(x)&=\left[ \sum _{i=1}^{r}h_i(q(t) )[A_ix(t)+B_iu(t)]\right] ^TP(x(t))\\&+(x(t))^TP\left[ \sum _{k=1}^{r}h_i(q(t) )[A_ix(t)+B_iu(t)]\right] \end{aligned} \end{aligned}$$

26 $$\begin{aligned} \begin{aligned} \dot{V}(x)&=\left[ \sum _{i=1}^{r}h_i(q(t) )A_ix(t)+\sum _{i=1}^{r}h_i(q(t) )B_iu(t)\right] ^TP(x(t))\\&+(x(t))^TP\left[ \sum _{k=1}^{r}h_i(q(t) )A_ix(t)+\sum _{i=1}^{r}h_i(q(t) )B_iu(t)\right] \end{aligned} \end{aligned}$$

The control law, *u*(*t*), is

27 $$\begin{aligned} u(t)=\sum _{k=1}^{r}h_k(q(t))T_kx(t) \end{aligned}$$

From equation (27), we can obtain

28 $$\begin{aligned} \begin{aligned} \dot{V}(x)&=\left[ \sum _{i=1}^{r}h_i(q(t) )A_ix(t)+\sum _{i=1}^{r}h_i(q(t) )B_i\sum _{k=1}^{r}h_k(q(t))T_kx(t)\right] ^TP(x(t))\\&+(x(t))^TP\left[ \sum _{k=1}^{r}h_i(q(t) )A_ix(t)+\sum _{i=1}^{r}h_i(q(t) )B_i\sum _{k=1}^{r}h_k(q(t))T_kx(t)\right] \end{aligned} \end{aligned}$$

Based on the attributes of the membership function^27^

$$\begin{aligned} \sum _{i=1}^{r}h_i(q(t))=\sum _{k=1}^{r}h_k(q(t))=\sum _{i=1}^{r}h_i(q(t))\sum _{k=1}^{r}h_k(q(t))=1 \end{aligned}$$

Therefore,

29 $$\begin{aligned} \dot{V}(x)=[A_ix(t)+B_iT_kx(t)]^TP(x(t)) +(x(t))^TP[A_ix(t)+B_iT_kx(t)] \end{aligned}$$

Now,

30 $$\begin{aligned} \dot{V}(x)=(x(t))^T\left[ A_i+B_iT_k\right] ^TPx(t) +(x(t))^TP\left[ A_i+B_iT_k\right] x(t) \end{aligned}$$

31 $$\begin{aligned} \dot{V}(x)=(x(t))^T\left[ P\left[ A_i+B_iT_k\right] +\left[ A_i+B_iT_k\right] ^TP\right] x \end{aligned}$$

Assume

32 $$\begin{aligned} F_i=\left[ A_i+B_iT_k\right] \end{aligned}$$

Equation (32) is substituted into equation (31)

33 $$\begin{aligned} \dot{V}(x)=(x(t))^T\left[ P\left[ F_i\right] +\left[ F_i\right] ^TP\right] x \end{aligned}$$

According to Lyapunov stability, $PF_i+F_i^TP<0$; hence,

34 $$\begin{aligned} \left[ (x(t))^T(PF_i+F^T_iP)(x(t))\right] \le 0 \end{aligned}$$

Equation (34) can be substituted into equation (33), yielding

35 $$\begin{aligned} \dot{V}(x(t))\le 0 \end{aligned}$$

Every trajectory with $t \rightarrow \infty$ that approaches the equilibrium or x(t) is invariant under LaSalle’s invariance principle. Equation (35) reveals the uniform convergence as the time derivative Lyapunov function ($\dot{V}$) monotonically decreases.

## Experimental results and discussion

**Fig. 6:**
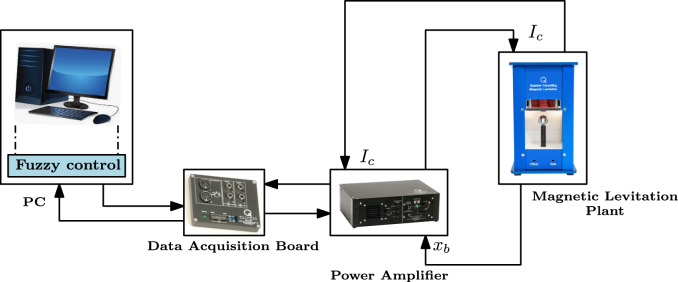
Experimental testbed of the maglev system.

Figure 6 illustrates an experimental setup of a maglev plant by Quanser^28^. The DAQ board can measure signals at 0.5 kHz with a 12 bit resolution at a input range of $\pm 10$V. The power amplifier regulates the current supplied to the electromagnet by delivering a output of $\pm 10$V at 3A. A QUARC control software interfacing with control algorithm developed in simulink is used for HIL validation. Using the PDC framework and LMI-based stability constraints, local state-feedback gains were constructed to meet the specified overshoot and settling time criteria. The controller synthesis was based on the developed T-S fuzzy model. The PIV controller gains are calibrated using a pole placement technique to achieve the requirements of 10% maximum overshoot and 2 sec settling time. The PIV gains are $K_p$= -227.23, $K_i$= -192.32, and $K_v$=-3.78, respectively. The maglev model contains a positive feedback loop, which results in negative controller gains. In an experimental setup, the feedforward controller gain, $K_{ff}$= 143.33A/m and the scale factor is set to 0.9 to account for the pull of gravity. The following test scenarios are used to assess the robustness and tracking capabilities control framework: (1) Nominal tracking control, (2) Disturbance rejection, and (3) Tracking against model uncertainties.The comparative study of results demonstrates the advantages of nonlinear control over linear control.

### Nominal tracking

**Fig. 7:**
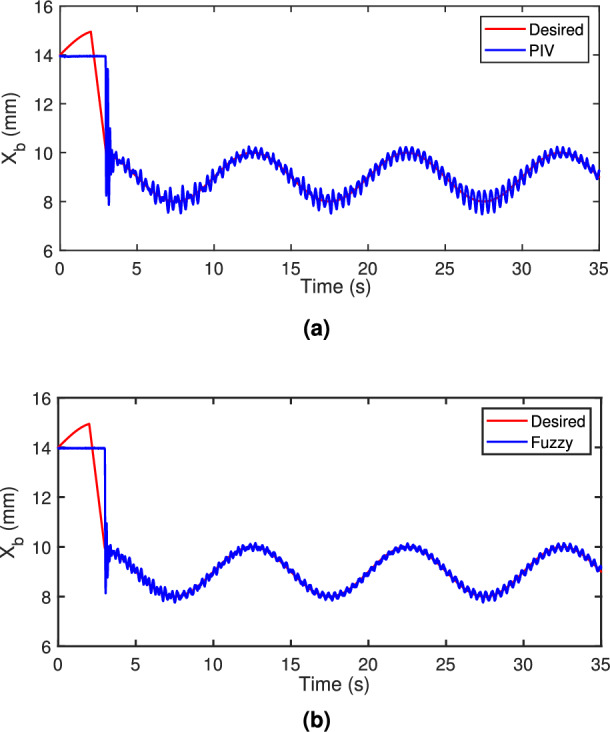
Trajectory tracking signal (**a**) PIV Controller (**b**) Fuzzy Controller.

**Fig. 8:**
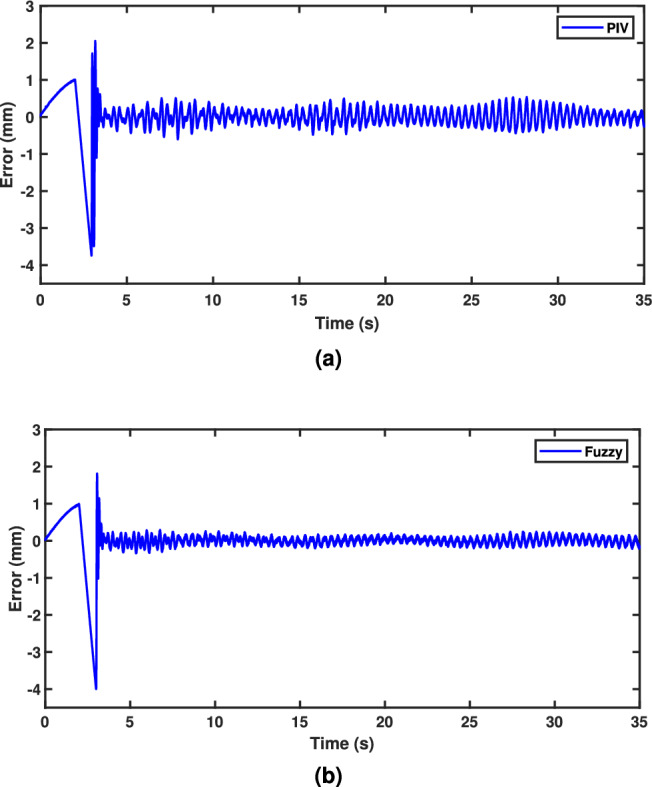
Tracking error (**a**) PIV Controller (**b**) Fuzzy Controller.

**Fig. 9:**
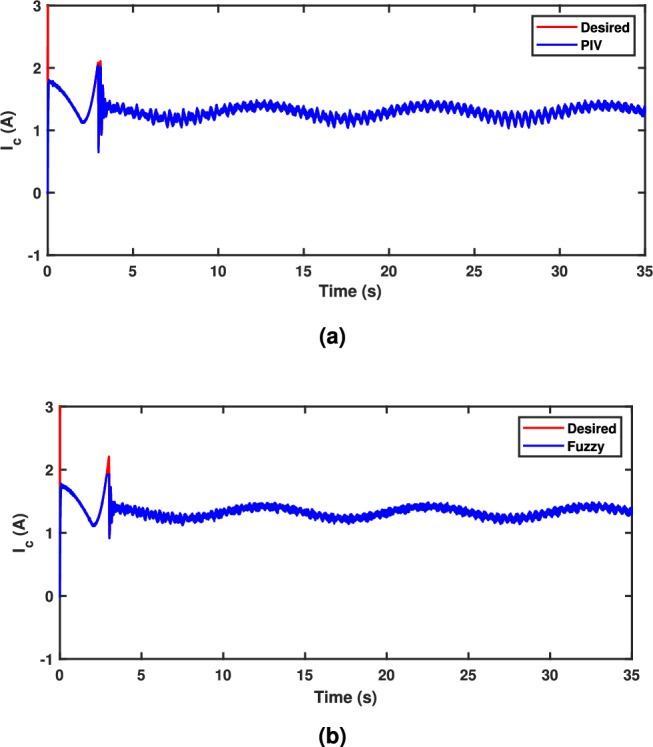
Coil Current Signal (**a**) PIV Controller (**b**) Fuzzy Controller.

**Fig. 10:**
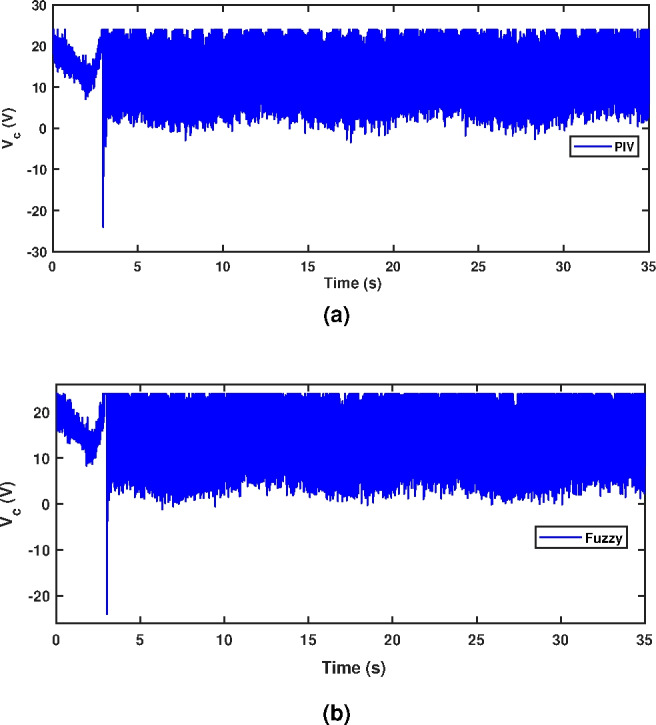
Control signal (**a**) PIV Controller (**b**) Fuzzy Controller.

**Fig. 11:**
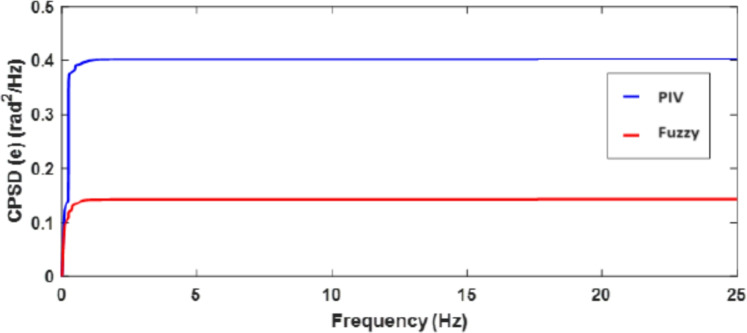
CPSD of tracking error.

A sinusoidal input with a 2 mm peak at 0.1 Hz frequency is used to assess the TS fuzzy approach’s path tracking ability. Figure 7 demonstrates the tracking response of both fuzzy and linear controllers. The fuzzy scheme greatly enhances tracking performance and enables smoother command following by minimizing the tracking error compared to the PIV controller. To assess the efficacy, the tracking error of PIV and fuzzy is illustrated in Fig. 8. It is evident that TS fuzzy control significantly reduces both tracking error and oscillations around the desired trajectory when compared to another control scheme that was taken into consideration for experimentation. Thus, the oscillation magnitude is 0.15 mm for fuzzy control and 0.8 mm for PIV control. The coil voltage and current for PIV and fuzzy methods are shown in Figs. 9 and 10. One can notice that fuzzy improves coil current precision by regulating the voltage. Figure 11 presents the CPSD of tracking error, which validates tracking performance in transient and steady-state over the frequency spectrum. The CPSD plot shows that the TS fuzzy control is superior to the PIV controller in terms of trajectory tracking performance with low vibrations.

### Disturbance rejection

The following pulse disturbance is introduced in the second test, which evaluates the regulatory performance of the fuzzy control technique.

36 $$\begin{aligned} d={\left\{ \begin{array}{ll} 0 & t<{12}\\ 0.2 & 12<t<16.75\\ 0 & 16.75<t \end{array}\right. } \end{aligned}$$

**Fig. 12:**
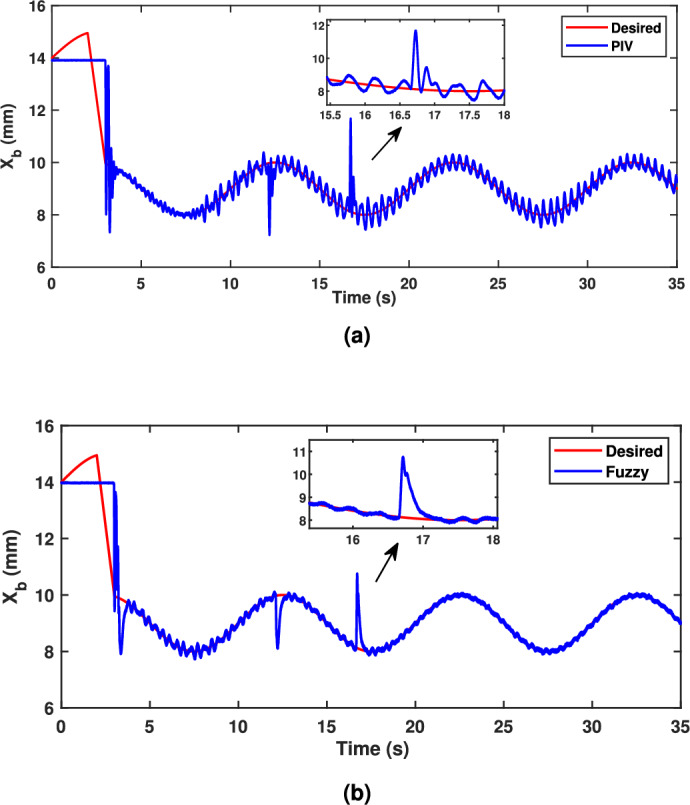
Tracking performance with disturbance rejection (**a**) PIV Controller (**b**) Fuzzy Controller.

From Figure 12, which depicts the regulatory response of PIV and fuzzy control techniques, where the fuzzy handles the ball position precisely from the beginning of the task execution. During an external disturbance, fuzzy control results in an efficient reduction of the oscillation amplitude deviation in the ball position. Even if the fuzzy gives an oscillating behaviour before the disturbance event, it performs better than the PIV controller at handling disturbances and bringing the ball position to a steady state more accurately by using precise fuzzy rules.

### Model uncertainty

**Fig. 13:**
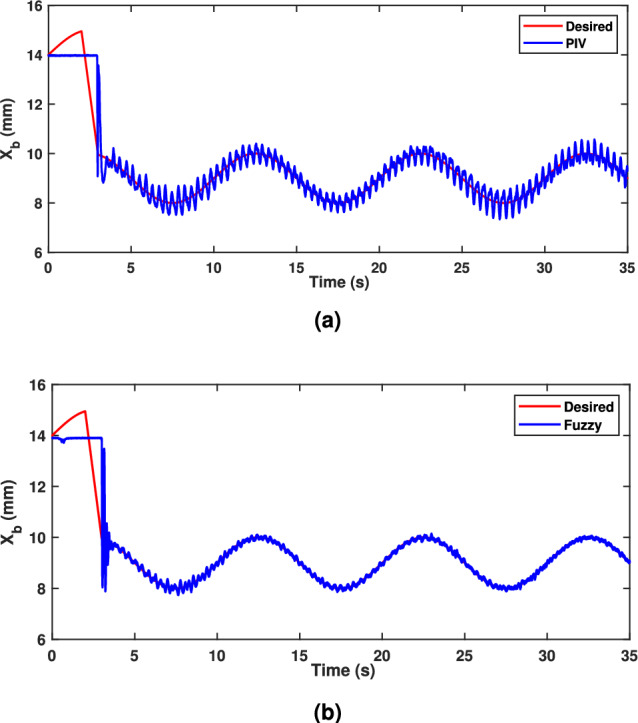
Tracking performance with model uncertainty (**a**) PIV Controller (**b**) Fuzzy Controller.

**Fig. 14:**
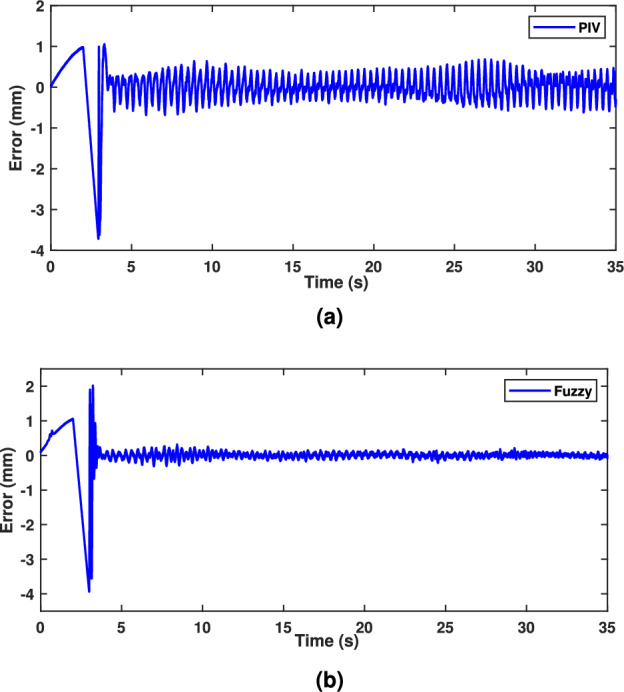
Tracking error (**a**) PIV Controller (**b**) Fuzzy Controller.

The robustness of the T-S fuzzy framework is further assessed via introducing perturbations and parametric variations. The force applied to the levitated object relies on the strength of the magnetic field. Higher levitation forces are usually the result of elevating the magnetic field strength. Hence, to investigate the fuzzy tracking control ability and robustness of nonlinear dynamical system under the occurrence of parametric uncertainties, the electromagnet coil resistance is subjected to a 10% uncertainty. Figures 13 and 14 illustrate the experimental ball position responses and tracking errors for both controllers. Although the feedback control system can handle certain model variations, it requires manual parameter re-tuning before implementing them into the controlled process, and it does not utilize error data to enhance the subsequent control effort for increased robustness. It is clear from Fig. 13 that fuzzy control, as compared to the PIV controller, substantiates higher tracking performance because it can capture the dynamics of the system from input and output data with fewer rules. Fuzzy control model dynamics create substantial oscillations in reaction to system uncertainties at first, but it gradually enables the ball to follow the command with less oscillation deviations.

### Performance measure

Table 4 compares the RMSE values for the PIV control approach and the proposed strategy to quantify the tracking performance for both test cases.. The T-S fuzzy system significantly improves tracking capabilities and robustness against external disturbances and parameter uncertainty. Furthermore, the performance criteria for both control strategies are shown in Table 5 in terms of IAE, ISE, ITAE, and ITSE. Through improvements in tracking performance and robustness over PIV control scheme, the results showed the significant effectiveness of the TS fuzzy control strategy. One notable difference between this study and the PIV approach is the fuzzy scheme takes into account the maglev plant’s nonlinearity and provides a superior trajectory that ensures both tracking and robustness properties. The tracking metrics of the proposed strategy are compared with those of similar control schemes detailed in the literature on maglev systems in Table 6.

**Table 4:** RMSE values of the test cases.

Controllers	Test Cases		
	NT	DR	MU
PIV	0.5673	0.5689	0.6348
Fuzzy	0.3790	0.4133	0.3911

**Table 5:** Performance Metrics for the test cases.

Metrics	NT		DR		MU	
	PIV	Fuzzy	PIV	Fuzzy	PIV	Fuzzy
IAE	0.0416	0.0321	0.0733	0.0365	0.0849	0.0116
ISE	0.0131	0.0025	0.0157	0.0039	0.0171	0.0027
ITAE	0.1375	0.1067	0.6547	0.4836	0.7823	0.1325
ITSE	0.0054	0.0013	0.3958	0.1079	0.0237	0.0038

**Table 6:** Comparison of trajectory tracking RMSE values with other methods.

References	Controller	RMSE (in mm)
Lin et al. 2007^14^	Backstepping Control	0.58
Lin et al. 2009^13^	Sliding Mode Control	0.55
Bächle et al. 2013^15^	MPC	0.48
Proposed	PIV	0.567–0.635
Proposed	PDC-TS Fuzzy	0.379–0.413

## Conclusion

This paper proposed a PDC-TS fuzzy system to improve the trajectory tracking performance of a magnetic levitation (maglev) system and control its nonlinear behaviour. To reduce rapid changes in the controlled variable, the method combines a velocity compensation mechanism with an adaptive fuzzy feedback gain state-feedback controller. The Lyapunov direct approach and LMIs were used to show the T-S fuzzy control’s asymptotic stability. Experiments and hardware-in-the-loop (HIL) testing on a maglev benchmark system demonstrated that the proposed T-S fuzzy control can offer better tracking and stabilization than a linear control strategy, especially when there are external disturbances and changes in the parameters. For nonlinear and unstable systems, the proposed T-S fuzzy technique provides clear advantages over traditional controllers. It offers robustness against changes in parameters and external disturbances, lessens the need of precise modeling, and employs velocity correction to reduce the effects of sensor noise. However, these results show significant performance improvements, they are specific to the setup and conditions that were tested. For example, the stability and performance of the system may be affected by environmental conditions that were not specifically taken into account, such as wind, temperature, humidity, and changes in coil currents or magnet configurations. Additionally, the proposed strategy was verified on a particular experimental platform, maglev, providing a controlled proof of concept. However, more testing on various configurations would be required to verify wider applicability. The study could overcome these constraints by investigating the best fuzzy logic controllers using meta-heuristic optimization techniques, implementing system identification techniques to increase model accuracy, and integrating reinforcement learning-based fuzzy control to handle unknown parameters. Expanding the study to include a wider variety of system configurations and operating situations would confirm the method’s robustness and suitability proposed method can be thoroughly examined.

## Data Availability

All data generated or analysed during this study are included in this published article [and its supplementary information files].
